# Sulfamethoxazole Levels in HIV-Exposed Uninfected Ugandan Children

**DOI:** 10.4269/ajtmh.17-0933

**Published:** 2018-04-23

**Authors:** Jingo Kasule, Erin E. Gabriel, Aggrey Anok, Jillian Neal, Richard T. Eastman, Scott Penzak, Kevin Newell, David Serwadda, Patrick E. Duffy, Steven J. Reynolds, Charlotte V. Hobbs

**Affiliations:** 1 Rakai Health Sciences Program, Kalisizo, Uganda;; 2 Division of Intramural Research, National Institute of Allergy and Infectious Diseases, National Institutes of Health, Bethesda, Maryland;; 3 Department of Medical Epidemiology and Biostatistics, Karolinska Institutet, Stockholm, Sweden;; 4 Division of Preclinical Innovation, National Center for Advancing Translational Sciences, National Institutes of Health, Bethesda, Maryland;; 5 College of Pharmacy, University of North Texas, Fort Worth, Texas;; 6 Clinical Research Directorate/Clinical Monitoring Research Program, Leidos Biomedical Research, Inc., National Cancer Institute Campus at Frederick, Frederick, Maryland;; 7 School of Public Health, Makerere College of Health Sciences, Kampala, Uganda;; 8 University of Mississippi Medical Center, Batson Children’s Hospital, Jackson, Mississippi

## Abstract

Trimethoprim–sulfamethoxazole (TMP–SMX) prophylaxis in HIV-uninfected, exposed (HUE) children variably reduces clinical malaria burden despite antifolate resistance, but data regarding achieved serum levels and adherence are lacking. Serum samples from 70 HUE children aged 3–12 months from Rakai, Uganda, enrolled in an observational study were assayed for random SMX levels using a colorimetric assay. Adherence with TMP–SMX prophylaxis data (yes/no) was also collected. Of 148 visits with concurrent SMX levels available, 56% had self-reported adherence with TMP–SMX therapy. Among these 82 visits, mean (standard deviation) level was 19.78 (19.22) µg/mL, but 33% had SMX levels below half maximal inhibitory concentrations (IC50) for *Plasmodium falciparum* with some, but not all, of the reported antifolate resistance mutations reported in Uganda. With TMP–SMX prophylaxis, suboptimal adherence is concerning. Sulfamethoxazole levels below IC50s required to overcome malaria parasites with multiple antifolate resistance mutations may be significant. Further study of TMP–SMX in this context is needed.

## INTRODUCTION

Malaria is highly prevalent in many areas of the world where HIV-infected children live, especially sub-Saharan Africa. Studies have shown that when HIV and malaria are present as coinfections, each disease can enhance the pathogenicity of the other.^1^ Moreover, as more patients are managed for HIV infection in malaria-endemic areas, understanding the impact of drugs used in HIV exposure and infection on malaria infection is important.

The World Health Organization recommends daily trimethoprim–sulfamethoxazole (TMP–SMX) prophylaxis for children of HIV-infected mothers daily starting at 4–6 weeks of age and continued until HIV infection has been excluded by an age-appropriate HIV test, after cessation of breastfeeding.^2^ Many HIV-uninfected, exposed (HUE) children in sub-Saharan Africa reside in malaria-endemic areas. Trimethoprim–sulfamethoxazole has been shown to have varying degrees of antimalarial impact even in the face of high antifolate resistance prevalence,^3,4^ but few studies have examined that TMP–SMX achieved levels and adherence in these populations. Herein, we report SMX levels (as a component of TMP–SMX) and adherence in a population of HUE children in an observational study.

## METHODS

Infants aged 0–12 months from Rakai district were eligible for enrollment and followed up at the Kalisizo Hospital and Rakai Health Services Program. Subjects were followed up monthly from February 2015 to August 2015. The original study design included enrollment of HUE and HIV-uninfected, unexposed children (HUU) with a primary objective of characterizing malaria incidence, but was stopped early for futility because of low malaria incidence in the region. This report therefore presents data from an exploratory objective. No drug was administered as part of this observational study, but children received standard-of-care management in Uganda, which for children of HIV-infected mothers involves daily nevirapine (NVP) or azidothymidine (AZT) from birth through 4–6 weeks of age, regardless of infant feeding method, along with daily prophylactic doses of TMP–SMX starting by 6 weeks of age and continuing until 6 weeks after breastfeeding is discontinued.^2,5^ Children of HIV-infected mothers were breastfed, as per World Health Organization (WHO) recommendations, up to at least 12 months of life,^5^ and HIV tests were performed after birth and once after the cessation of breastfeeding (HIV exposure) at Kalisizo hospital as per standard of care.^2,5^ Mothers of HUE children also received standard of care “Option B+,” which entails treating the mother with triple antiretroviral therapy (ARV) as soon as she is diagnosed and continuing for life, regardless of CD4 count. Triple ARV treatment refers to the use of one of the recommended three-drug fully suppressive treatment options, including AZT, NVP, or lamivudine.^6^

At study visits, history and physicals were performed, TMP–SMX adherence data (yes/no) were collected (with “reported adherence” for those guardians who responded “yes”; “reported nonadherance” for those guardians responding “no”; or “missing self-report” for data not obtained at that visit), and heel/finger stick and venous blood collected for drug levels (SMX of TMP–SMX). To preserve sample stability, the samples were stored at −80°C immediately after collection and processing on site in Uganda. The samples were shipped back on dry ice (with temperature monitoring and no thawing). Once received, the samples were immediately stored at −80°C until use for the assay. If any samples needed to be rerun, that sample was kept at −20°C after the initial run and until use for the next run.

At all visits, children were tested for malaria using Giemsa-stained malaria thick smear. Clinical illness was managed according to Integrated Management of Childhood Illness guidelines^7^ and WHO malaria treatment recommendations.^8^ Dried blood spots were also collected for malaria polymerase chain reaction, performed as previously described.^9^ Maternal HIV status was determined from documented medical history. All subjects received insecticide-treated bednets to prevent malaria if they did not already have them.

### Study site.

Rakai district registers an HIV seroprevalence of 8.5% among the pregnant mothers across the various geographical populations, with an estimated 2,000 births per year (F. Nalugoda, personal communication). Rakai district is on a plateau at an altitude ranging between 750 and 900 m and has fair rainfall throughout the year, with relatively dry periods during January and February and from June through August. Peak rainfall varies from year to year, but occurs typically in March/April and October/November.^10^ Malaria is meso- to holoendemic with year-round transmission and highest intensity after the rainy seasons or in communities adjacent to lakes and other mosquito breeding sites.^11^

### Assay for sulfa level.

Sulfa levels in serum were measured using a previously described colorimetric assay^12^ with some modifications, including adaptation to 96-well plates. Briefly, 20 μL of serum was diluted in 260 μL of water, incubated at room temperature for 5 minutes, and boiled (98°C) for 1 hour in a thermocycler to deacetylate the small portion of SMX, which is naturally acetylated in the body. The plate was then centrifuged at 2,000 rpm for 3 minutes. A 120-μL quantity of 20% *p*-toluenesulfonic acid in 0.2 M HCl was added to each sample and incubated for 5 minutes to precipitate serum proteins. The plate was then centrifuged at 4,000 rpm for 10 minutes. A 100-μL quantity of supernatant was recovered and combined with 20 μL of a citric acid buffer, followed by 40 μL of a 2% dimethylaminobenzaldehyde solution in ethanol, resulting in a color change that was quantified at 450 nm as a measure of sulfa levels. The assay was standardized using control serum, to which SMX of known level was added in serial dilutions. Our limit of detection was 1.9 μg/mL and, therefore, levels equal to 1.9 μg/mL or less were considered undetectable. Standard curves were included with each plate to allow for determination of sample level. Samples with values above 5.8 μg/mL were considered therapeutic for IC50 for parasite strains in this regions, based on in vitro growth inhibition assay IC50 data for SMX with *Plasmodium falciparum* strains with known antifolate resistance mutations^13^ and known resistance mutations of F32 and K1 compared with resistance data published for Uganda, indicating a range of 2.53–5.8 μg/mL.^13–15^

### Statistics.

R version 3.3.1 was used for all calculations, including summary statistics (means, standard deviations [SDs], and percentages).

### Ethics.

The study was approved by the Uganda Virus Research Institute Research and Ethics Committee, the Uganda National Council for Science and Technology, and the National Institute of Allergy and Infectious Diseases Intramural Institutional Review Board. Parents or guardians of infants and children enrolled provided written consent for the infants enrolled in this study.

## RESULTS

### Demographics.

Seventy HUE subjects were enrolled, aged 3–12 months while on study with a mean duration on study of 11.78 weeks (SD 6.84). Mean age at enrollment was 6.76 (2.37) years (Table 1). Demographics, malaria incidence, breastfeeding, and bednet use for HUE subjects are also summarized in Table 1. Parallel data for the HUU group are presented in Supplemental Table 1.

**Table 1:** Demographic information and malaria episodes for HIV-uninfected, exposed (HUE) children enrolled on study

Category	Subcategory	Subjects (*N* = 70)
Age at enrollment (month)	Mean (SD)	6.76 (2.37)
	Range	[3, 12]
Duration on study (week)	Mean (SD)	11.78 (6.84)
	Range	[0, 24]
Age group (month)	3–4	14 (20%)
	5–8	36 (51.4%)
	9–12	20 (28.6%)
Gender	Female	40 (57.1%)
	Male	30 (42.9%)
Malaria episodes	PCR	2 (0.71%)*
Breastfeeding† (age group in months)	3–5	16 (84.21%)
	6–12	41 (89.13%)
Bednet use†	–	66 (94.29%)

SD = standard deviation.

* Two total positive PCRs in two unique HUE subjects, of 281 samples analyzed, of 499 samples analyzed for this group (780 for the whole study).

† The figures indicated represent subject responses at their last study visit.

### TMP–SMX adherence data and sulfa levels.

**Figure 1.**
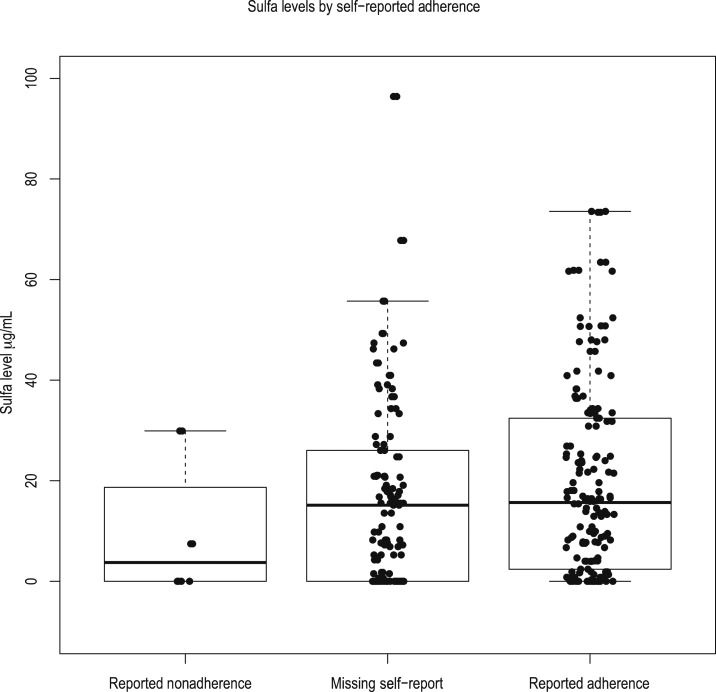
Among clinical visits, 56% reported adherence with TMP–SMX therapy (*N* = 82). Among these visits, the mean drug level was 19.78 (SD 19.22) µg/mL; yet, 33% of concurrent drug levels were below IC50s for *Plasmodium falciparum* with some, but not all, of the reported antifolate resistance mutations recently reported in these areas. SD = standard deviation. This figure appears in color at www.ajtmh.org.

Among 148 clinical visits for which samples were obtained and assayed for concurrent SMX levels, only 56% had self-reported adherence with TMP–SMX therapy. Among these 82 visits, the mean concurrent drug level was 19.78 (SD 19.22) µg/mL. As well, 33% of concurrent drug levels were below 5.8 μg/mL (IC50 range 2.53–5.8 μg/mL^13–15^) (Figure 1).

## DISCUSSION

**Figure 2.**
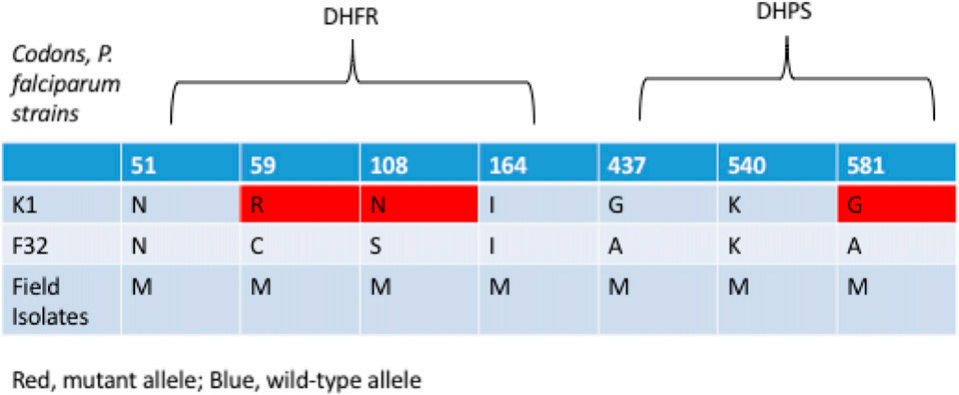
Resistance mutations of antifolates in laboratory-adapted strains share common resistance mutations with field strains reported in Uganda.^13–15^ This figure appears in color at www.ajtmh.org.

In our study, adherence to TMP–SMX was reported in only 56% of clinical visits. Even when adherence with TMP–SMX therapy was reported, 33% of concurrent drug levels of SMX were below IC50s for *P. falciparum* with some, but not all, of the already reported antifolate resistance mutations that exist in Uganda (Figure 2).

Previous studies have shown over time that HIV-infected and exposed children on TMP–SMX prophylaxis have reduced clinical malaria burden, and the degree of the effect likely depends on transmission intensity and preexisting antifolate resistance mutation prevalence in the region.^3,4^ Few prior studies have examined TMP–SMX levels in this context.^16^ A recent study of 136 West African children on ARV and TMP–SMX prophylaxis suggested that overall TMP–SMX levels in children dosed according to WHO recommendations were lower than those achieved in adults,^17^ although the relevance of this to preventing infections or driving drug resistance requires further study. Trimethoprim–sulfamethoxazole prophylaxis impact on the development of malaria-specific immunity in children requires further study. Trimethoprim–sulfamethoxazole also has activity that surpasses expected antimicrobial effects in bacterial and fungal infections in HIV-exposed and infected patient populations.^3^ Both in malaria and other infections, TMP–SMX prophylaxis studies should include more extensive drug-level assessment as a reflection of reported adherence, especially given how widely it is now being used.

Weaknesses of our study include that we were not able to perform more detailed pharmacokinetics of TMP–SMX on these children. In addition, we acknowledge certain caveats in attempting to interpret available in vitro *P. falciparum* study data for antifolates to clinical efficacy. First, we know that in vitro, indications of antifolate resistance do not directly translate to clinical failure—one reason being that in vitro assays are not able to account for the host immunity, which in malaria, is associated with control of drug-resistant parasite strains.^18^ Moreover, interpretation of *P. falciparum* parasite strain in vitro susceptibility to antifolate medications in general is well known in its complexity because of the impact that exogenous folate supplementation can have on varying parasite strains.^19^ In parallel, we know that patients will have varying degrees of underlying nutrition, which makes direct extrapolation of these assays more difficult. And although many other studies have examined TMP–SMX impact on malaria in children,^3,4^ we did not observe robust transmission in either group to draw any comparable conclusion in this study (Table 1, Supplemental Table 1). However, with all those caveats taken into account, it is still concerning to note that if we further adjusted our random SMX levels to account for the amount of protein-bound (70%) as opposed to free (active) drug (30%), levels are considerably lower than IC50s.^20^

Although TMP–SMX was not originally intended to treat or provide prophylaxis against malaria, the HIV pandemic in areas of malaria endemicity presents continued questions surrounding TMP–SMX impact on malaria. Further studies are required.

## Supplementary Material

Supplemental Table.
