# Supplementary material for: Sulfamethoxazole Levels in HIV-Exposed Uninfected Ugandan Children
Source: Am J Trop Med Hyg. 2018 Apr 23;98(6):1718–21. doi: 10.4269/ajtmh.17-0933 (PMC6086194; doi:10.4269/ajtmh.17-0933)
Supplement: Supplementary file 1 [file tpmd170933.SD1.pdf]

Supplemental Table 1

Demographic information and malaria episodes for HIV-uninfected, unexposed (HUU) children enrolled on study

Category	Subcategory	Cohort 2 (*N* = 100)
Age at enrollment (month)	Mean (SD)	5.94 (2.78)
	Range	[2, 10]
Duration on study (week)	Mean (SD)	15.94 (6.52)
	Range	[0, 24]
Age group (month)	2–4	43 (43%)
	5–8	28 (28%)
	9–10	29 (29%)
Gender	Female	47 (47%)
	Male	53 (53%)
Malaria episodes	PCR	7* (1.4%)
Breastfeeding† (age group in months)	2–5	47 (95.92%)
	6–12	40 (93.02%)
Bednet use†	–	95 (95%)

SD = standard deviation.

*Seven total positive PCRs in six unique HUU subjects, of 499 samples analyzed for this group (780 for the whole study): this PCR did not account for new infections compared with recrudescent infections; however, the visits for the same subjects with two positive results occurred 28 days apart.

†The figures indicated represent subject responses at their last study visit.
